# Cardiorespiratory fitness as a mediator in the relationship between lung function and blood pressure in adults

**DOI:** 10.1590/1414-431X2022e11754

**Published:** 2022-07-25

**Authors:** F.R. Almeida, T.L.V.D.P. Ostolin, V.R. Almeida, B.B. Gonze, E.F. Sperandio, M.S.M.P. Simões, I. Godoy, S.E. Tanni, M. Romiti, R.L. Arantes, V.Z. Dourado

**Affiliations:** 1 Departamento de Ciências do Movimento Humano, Universidade Federal de São Paulo, Santos, SP, Brasil; 2 Disciplina de Pneumologia do Departamento de Clínica Médica da Faculdade de Medicina de Botucatu, Universidade Estadual Paulista, Botucatu, SP, Brasil; 3 Angiocorpore Instituto de Medicina Cardiovascular, Santos, SP, Brasil; 4 Lown Scholars Program, Harvard T.H. Chan School of Public Health, Boston, MA, USA

**Keywords:** CRF, Spirometry, V̇O_2_, Hypertension, Mediation analysis

## Abstract

It is unclear whether physical activity and cardiorespiratory fitness (CRF) are pathways that link low pulmonary function (LPF) to increased blood pressure (BP). Therefore, we investigated the extent to which CRF and moderate-to-vigorous physical activity (MVPA) mediate the relationship between LPF and high BP in adults. We conducted a cross-sectional study with 1,362 participants that underwent cardiopulmonary exercise testing (CPET), spirometry, and wore an accelerometer to determine physical activity patterns. We performed mediation analyses using structural equations considering peak oxygen uptake (V̇O_2_) and MVPA as mediators, forced vital capacity (FVC) and forced expiratory volume in the first second (FEV1) as independent variables, and systolic and diastolic blood pressure (SBP, DBP) as dependent variables. The probability of alpha error was set at 5%. We found a significant total effect of FVC on SBP and DBP considering V̇O_2_ as mediator (P<0.01). Indirect effects were also significant, with 42.6% of the total effect of FVC on SBP and 77% on DBP mediated by V̇O_2_ (P<0.01). We did not observe a direct effect of FVC on SBP and DBP. Considering FEV1 as an independent variable, the total effect on SBP was also significant, as were the indirect effects, mediated by V̇O_2_ at 14.8% for SBP and 7.6% for DBP (P<0.01). We did not find an indirect effect of FVC or FEV1 considering the MVPA as a mediator. CRF mediates the pathway that links LPF and elevated BP. Therefore, CRF is more sensitive to variations in FVC and FEV1 than MVPA.

## Introduction

Cardiovascular disease is the leading cause of death worldwide, followed by pulmonary diseases and respiratory infections (1). Among the risk factors for cardiovascular disease, elevated blood pressure (BP) is a prevalent condition in adults and may be affected by biological and lifestyle variables, including physical activity level and fitness (2,3). Similarly, low peak lung function is common even in early adulthood (4). Thus, monitoring lung function may allow screening subjects with increased risk of early respiratory, cardiovascular, and metabolic abnormalities and premature death (4). However, both elevated BP and low lung function are often silent conditions, being frequently undiagnosed due to the absence of clinical symptoms (3,5), although they can be detected by simple and inexpensive assessment tools.

Lung function and cardiorespiratory fitness (CRF) affect BP. There are reports of elevated BP related to lung disorders and lung function decline (6,7). In addition, previous studies have shown the association between spirometric indices, e.g., forced vital capacity (FVC) and BP (6). Likewise, low CRF is associated with elevated BP. Moreover, the literature widely advocates the cardioprotective role of CRF (8,9).

Among lifestyle variables that affect BP, there is consistent evidence supporting that physical inactivity (i.e., to perform less than 150 min per week of moderate-to-vigorous physical activity - MVPA) is associated with a higher incidence of cardiovascular diseases, including hypertension (10,11). Studies such as the Nurses' Health Study II, the Aerobics Center Longitudinal Study, and Coronary Artery Risk Development in Young Adults showed that higher levels of self-reported daily physical activity and CRF are associated with a lower incidence of hypertension (11,12).

Despite the well-described relationship between lung function and BP, the possible pathways underlying this relationship are still unclear. Also, it is unknown to which extent this relationship is affected by CRF and physical activity level since all of these variables are associated with BP. To fulfill this gap and investigate the potential role of mediators in these relationships, mediation analysis is the appropriate method since it allows the exploration and quantification of potential cause-and-effect relationships (13,14). Although some of these associations have already been investigated through multivariate methods, the variables are often included as confounders instead of exploring their possible mediating role.

Since spirometric indices are associated with BP, CRF, and MVPA, and MVPA and CRF are also significantly correlated to BP, we hypothesized that CRF and MVPA mediate the relationship between lung function and BP. Therefore, we aimed to investigate if CRF and MVPA are mediators in the relationship between spirometric indices and BP in asymptomatic adults. Secondarily, we intended to investigate the extent of this mediation.

## Material and Methods

### Study design and participants

**Figure 1:**
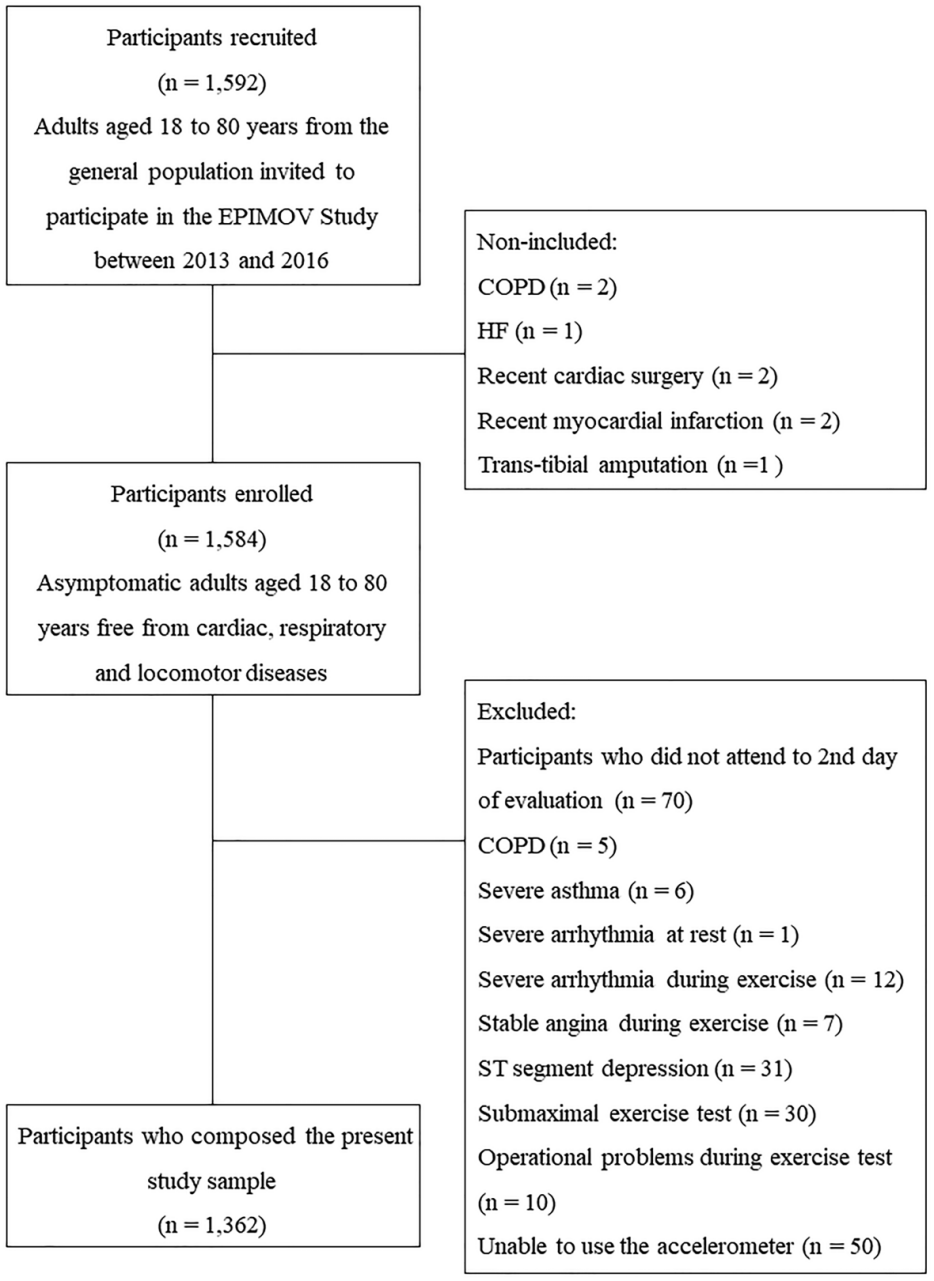
Flowchart of the study. COPD: chronic obstructive pulmonary disease; HF: heart failure; EPIMOV: Epidemiology and Human Movement Study.

We conducted a cross-sectional study with 1,362 asymptomatic adults eligible from the Epidemiology and Human Movement (EPIMOV) study (Figure 1). In brief, the EPIMOV study was a prospective cohort study that investigated the correlation between low physical activity level and low physical fitness and cardiorespiratory, metabolic, and locomotor outcomes over a short period of time. We recruited volunteers through advertisements in social media, local universities, and newspapers. Volunteer recruitment and data collection were conducted from 2013 to 2016. The Ethics Committee of the Federal University of São Paulo approved the study (#186796), and all volunteers signed an informed consent before participation.

The inclusion criteria of the EPIMOV study were asymptomatic subjects or subjects with treated and controlled chronic conditions of either sex and aged 18 to 80 years. Exclusion criteria were previously diagnosed pulmonary, cardiovascular, musculoskeletal, or neuromuscular diseases, recent respiratory infections, abnormalities during exercise testing (i.e., chest pain, sudden drop in diastolic blood pressure (DBP) ≥20 mmHg or systolic blood pressure (SBP) ≥250 mmHg, severe arrhythmia, stable angina, ST-segment depression), or early interruption of cardiopulmonary exercise testing (CPET) for reasons other than exhaustion (i.e., submaximal exercise, operational problems during the test). Participants who demonstrated spirometric indices suggestive of obstructive pulmonary disease or severe asthma and those who did not attend the second day of evaluation or were unable to use the accelerometer were also excluded (Figure 1).

In the EPIMOV study, we carried out the study protocol on three separate days, respectively at the Angiocorpore Institute of Cardiovascular Medicine, a clinical analysis laboratory, and the EPIMOV laboratory. On the first day, we collected clinical and sociodemographic information (self-report), anthropometric measurements (scale with stadiometer), lung function (spirometry), followed by CRF (CPET). On the second day, we evaluated the lipid and glycemic profile (blood sample analysis). On the third day, seven days after the initial evaluation, participants returned the triaxial accelerometer and we assessed body composition (bioelectrical impedance), heart rate variability (heart rate monitor), postural balance (force platform), muscle function (isokinetic dynamometry and handgrip strength), functional exercise capacity (6-min walking test), cardiovascular risk score (self-report), and BP (electronic sphygmomanometer). We provided a detailed description of measurements that were included in the present study, as described below.

### Main outcome: blood pressure

In a seated position, SBP and DBP were measured after five min of rest using a validated digital device (Omron HEM 705CPINT, USA) (15). We recorded three measurements with a 1-min interval between them. The average of the last two measurements was considered for analysis. All blood pressure measurements were taken during the morning (from 8 a.m. to 10 a.m.), when the blood pressure reaches a plateau (16).

### Main predictors: forced vital capacity and forced expiratory volume in the first second

Lung function was assessed using a portable spirometer (Quark PFT, COSMED, Italy) during the morning or afternoon according to the participant's convenience to carry out the assessments. According to the American Thoracic Society recommendations (17), we registered FVC, forced expiratory volume in the first second (FEV_1_), and the FEV_1_/FVC ratio. For those who had an FEV1/FVC <0.7 on pre-bronchodilator spirometry, we conducted forced spirometry 15 min after the patient inhaled 400 μg of salbutamol. We considered a spirometrically-defined restrictive ventilatory defect in the presence of FVC <80% of the predicted value with an FEV_1_/FVC ratio of ≥70% (18,19).

### Potential mediators

#### Cardiorespiratory fitness

We conducted the CPET on a treadmill (ATL, Inbrasport, Brazil) following a ramp protocol in which increases in speed and inclination were individualized according to the estimated maximum peak oxygen uptake (V̇O_2_) (20,21). We performed the tests for all participants at the same altitude, atmospheric pressure, and temperature (22°C), and a cardiologist supervised all tests. CPET was performed after spirometry in the morning or afternoon.

The maximal CPET aims to take the subject to exhaustion within 8 and 12 min of exercise. Before the test started, the subjects rested for 3 min, allowing an initial evaluation of baseline measurements. A speed of 3 km/h and an incline of 0% were set for all participants at the beginning of the test. When the subjects reached exhaustion, the CPET was interrupted at the subject's request or the physician’s, if there were signs of myocardial ischemia (ST-segment depression), chest pain, sudden drop in DBP ≥20 mmHg or SBP ≥250 mmHg, signs of respiratory failure, loss of coordination, or mental confusion. The exercise was followed by 3 min of recovery.

The entire test was performed with a 12-lead electrocardiogram (C12X, COSMED). Every two min during the CPET, we assessed BP and perceived exertion regarding dyspnea and lower limb fatigue using the modified Borg scale.

Throughout the test, metabolic, cardiovascular, and ventilatory responses were measured breath by breath with a gas analyzer (Quark PFT, COSMED). The V̇O_2_ was measured breath by breath, and we calculated the average V̇O_2_ every 15 s. We considered as maximum effort those tests in which the subject achieved a heart rate at the peak of exercise ≥85% of the predicted value for age (220-age), a gas exchange rate (R) ≥1.0, or a V̇O_2_ plateau. The average V̇O_2_ of the last 15 s, immediately before the recovery phase, was considered the peak V̇O_2_ (20).

#### Accelerometer-based sedentary behavior and physical activity

Physical activity level was evaluated with a previously validated triaxial accelerometer (ActiGraph GT3X+, MTI, USA) (22,23). The participants completed seven consecutive days of assessment during waking hours, excluding showers and aquatic activities. To be considered valid, the days of data collection were required to have at least 10 h of continuous monitoring, starting at wake-up time. Non-wear time and the thresholds for the intensity of the physical activity were evaluated as previously described (23).

The measurements were calculated as h/day considering the total wear time and the number of calendar days of use, and the percentage of the entire time. Activity counts of all three axes (vertical, horizontal, and mediolateral) were measured. The thresholds for the intensity of physical activity were sedentary behavior as the number of min spent with <100 counts per minute (cpm) (i.e., measured accelerations stored at 1 Hz), which represents <1.5 METs of energy expenditure; light-intensity physical activity as <1,951 cpm and <3 METs; and MVPA as >1,951 cpm and ≥3 METs (23). To be physically inactive is to not engage in sufficient physical activity, especially MVPA, in order to ensure its benefits (24). Conversely, engaging in postures or activities (e.g., sitting for long periods and watching TV) that require minimal movement and low energy expenditure (<1.5 METs) is considered sedentary behavior (24). Therefore, participants who achieved less than 150 min/week of MVPA or less than 75 min/week of vigorous physical activity were considered physically inactive (25).

### Covariates

#### Clinical and sociodemographic assessment

We obtained age, sex, race, educational level, and cardiovascular risk through self-report. The risk factors for cardiovascular disease were older age (>45 years old in men and >55 in women), hypertension, diabetes or hyperglycemia, dyslipidemia or hypercholesterolemia, current smoking, and family history of premature coronary heart disease (i.e., myocardial infarction or sudden death in first-degree relatives) (26).

#### Cardiovascular risk score

We calculated the cardiovascular risk score as recommended by the Brazilian Cardiology Society (26). Briefly, this continuous score is reported as a percentage and is calculated based on sex, age, SBP, treatment for arterial hypertension, current smoking, diabetes, and body mass index (BMI). The higher the score, the higher the cardiovascular risk.

#### Anthropometric evaluation

We measured the height and weight of participants using a scale with a stadiometer based on standard methods (27). We calculated BMI in kg/m^2^ and defined obesity as a BMI ≥30 kg/m^2^ (27).

### Statistical analysis

Statistical analysis was performed using STATA, version 14 (StataCorp, USA). Initially, the data were analyzed descriptively. Continuous and categorical variables are reported as means±SD and frequency (percentage), respectively. We set the probability of alpha error at 5% for all analyses.

We used structural equation modeling frameworks to examine whether CRF and MVPA mediate the relationship between spirometric indices (FVC and FEV_1_) and BP (SBP and DBP). FVC and FEV_1_ served as independent variables, CRF and MVPA as mediators, and SBP and DBP as dependent variables. We also included age, sex, height, and a clustered cardiovascular risk score (sex, age, SBP, treatment for arterial hypertension, current smoking, diabetes, and BMI) (26) as covariates in all models. Finally, we adjusted the models for CRF when MVPA was considered the potential mediator and *vice-versa*. Since MVPA was evaluated as a mediator in the mediation model and the mediation analysis is a series of linear regressions, the models were fitted for the amount of MVPA as a continuous variable.

Accordingly, the main premises for mediation analysis were ensured (28): the independent causal variables (FVC and FEV_1_) have to correlate significantly with the outcome (BP) (significant total effect, path *c*); the independent variables (FVC and FEV_1_) have to correlate significantly with the mediator (CRF and MVPA) (path *a*); and the mediators (CRF and MVPA), in turn, have to correlate significantly with the dependent variable (BP) (path *b*).

**Figure 2:**
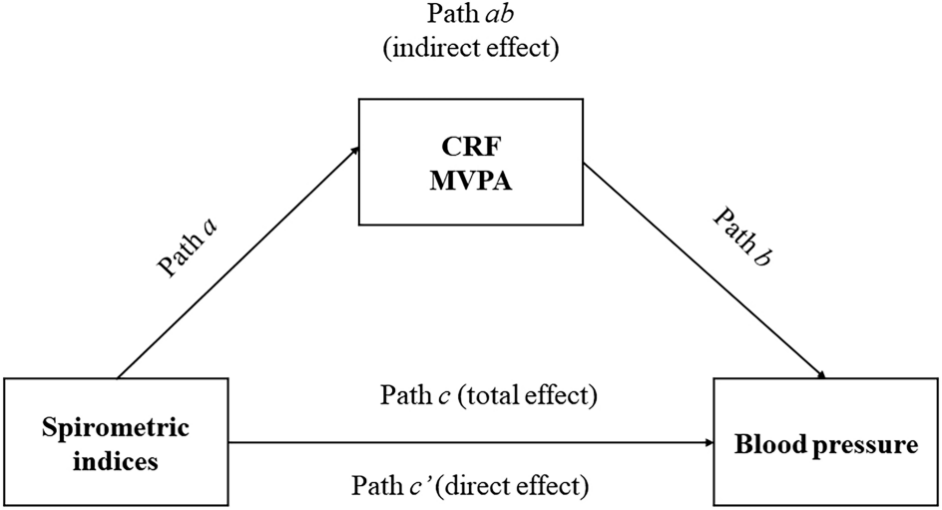
The structural equation modeling used in the present study for assessing the mediation role of cardiorespiratory fitness (CRF) and moderate-to-vigorous physical activity (MVPA) in the correlation between lung function and blood pressure.

As shown in Figure 2, we investigated the total (path *c*) and direct effects (path *c'*) using regression coefficients and significance between each model's independent and dependent variables. We also examined the indirect effect obtained from the product of coefficients (*a* × *b*, path *ab*).

The total effect *c* represents the correlation between spirometric indices and BP without considering the potential mediator (CRF or MVPA). In contrast, path *a* represents the correlation between the independent variables (FVC and FEV_1_) and the mediator (CRF or MVPA). Effect *b* represents the correlation between the mediator (CRF or MVPA) and the dependent variable (BP). After the necessary adjustment for confounders (29), the *ab* pathway represents the indirect effect of pulmonary function activity, i.e., the effect mediated by the CRF or MVPA. The mediated effect is simply the multiplication of effects *a* and *b*. Therefore, the direct effect *c'* represents the independent variable's effect after considering the mediated effect, i.e., *ab - c* (28). In the presence of a significant total effect (path *c*), the mediation is complete when the direct effect *c'* is zero and becomes non-significant after controlling for the mediator, which means that the effect of the independent variables (FVC and FEV_1_) on the dependent (BP) no longer exists when we consider the role of the mediator (CRF or MVPA).

Then, we calculated the percentage of the total effect mediated by the quotient *b/c*. As a general rule, we considered a complete mediation when the rate of the total mediated effect exceeds 80% (28). Conversely, in the case of the significant total effect, a partial mediation is considered when the direct effect remains significant and B≠0 after considering the mediator with percentages of total mediated effects (*ab/c*) below 80%.

In the present study, the structural equations were generated based on all available data (n=1362) using the full-information maximum likelihood, which allows valid inferences under the assumption that the data are missing at random.

Considering the influence of obesity on the spirometrically-defined restrictive ventilatory defect, we performed a sensitivity analysis stratifying our initial sample into obese (BMI ≥30 kg/m^2^) and non-obese subjects and conducted the mediation analysis. We found no significant differences, and therefore we presented the results of our entire sample here. Similarly, we found no differences when stratifying the sample by physical activity level (i.e., inactive and active subjects).

We evaluated the fit of the model with the comparative fit index, where we considered values above 0.9 as evidence of good fit. Finally, we used the Sobel-Goodman test to investigate the proportion of total mediated effects.

## Results

Participants were mainly overweight and physically active women with low prevalence of smoking and hypertension (Table 1).

**Table 1:** General characteristics of the study sample (n=1362).

Age (years)	46.7±14.2
Gender, %	
Males	39.7
Females	60.3
Weight (kg)	76.9±17.2
Height (m)	1.64±0.09
Body mass index (kg/m^2^)	28.4±6.0
Peak oxygen uptake (mL/min)	2385±881
Peak oxygen uptake (mL·min^-1^·kg^-1^)	32.1±11.6
Peak oxygen uptake (% pred.)	102.2±20.4
Moderate-to-vigorous physical activity (h/week)	4.66±2.89
Systolic blood pressure (mmHg)	127.7±16.4
Diastolic blood pressure (mmHg)	80.1±9.6
Cardiovascular risk, %	
Arterial hypertension^a^	19.9
Diabetes^a^	9.3
Dyslipidemia^a^	30.9
Obesity^a^	36.4
Current smoking^a^	11.0
Physical inactivity^b^	27.0
Framingham Cardiovascular Risk Score (%)	44.0±18.0
Spirometry	
FVC (% pred.)	95.3±13.3
FEV_1_ (% pred.)	94.8±14.0
VEF_1_/CVF (%)	81.3±6.0
Spirometrically-defined restrictive ventilatory disorder (%)	10

Data are reported as means±SD or as frequency and %. FEV_1_: forced expiratory volume in 1 s; FVC: forced vital capacity. ^a^Self-reported cardiovascular risk factor. ^b^Accelerometer-based physical inactivity.

The use of a triaxial accelerometer was, on average, 884±76 min per day. Despite being mostly physically active (>150 min MVPA/week), participants spent 73, 22, and 5% of the total accelerometer wear-time on sedentary behavior (i.e., <100 cpm, ≤1.5 MET), light-intensity physical activity, and MVPA, respectively.

**Figure 3:**
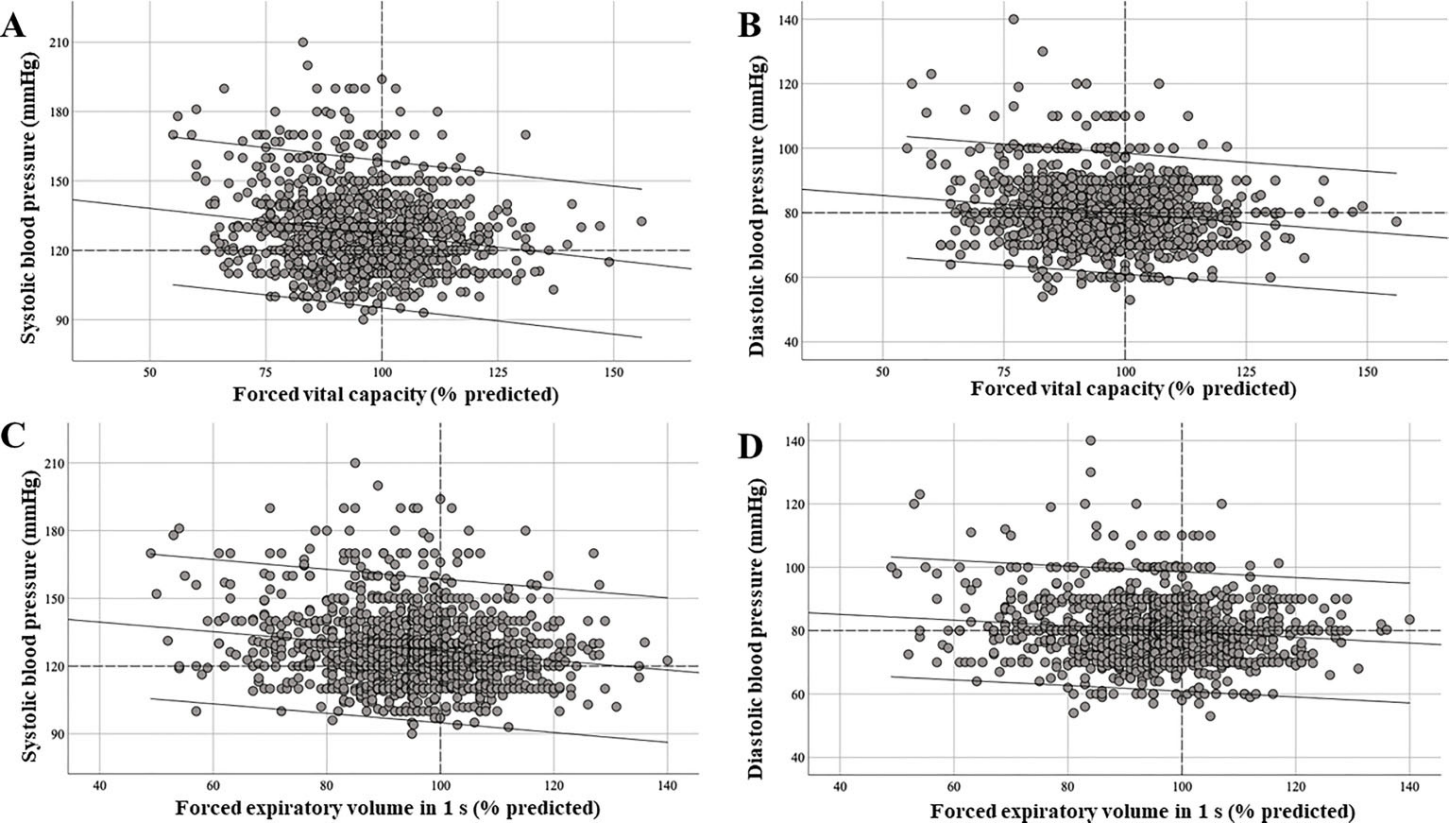
Correlations between lung function and blood pressure. (**A**) Forced vital capacity *vs* systolic blood pressure (r = –0.289; P<0.0001); (**B**) forced vital capacity *vs* diastolic blood pressure (r = –0.256; P<0.0001); (**C**) forced expiratory volume in 1 s *vs* systolic blood pressure (r = –0.269; P<0.0001); (**D**) forced expiratory volume in 1 s *vs* diastolic blood pressure (r = –0.203; P<0.0001).

We found significant negative correlations between FVC and SBP and DBP (Figure 3A and B) and between FEV1 and SBP and DBP (Figure 3C and D).

**Figure 4:**
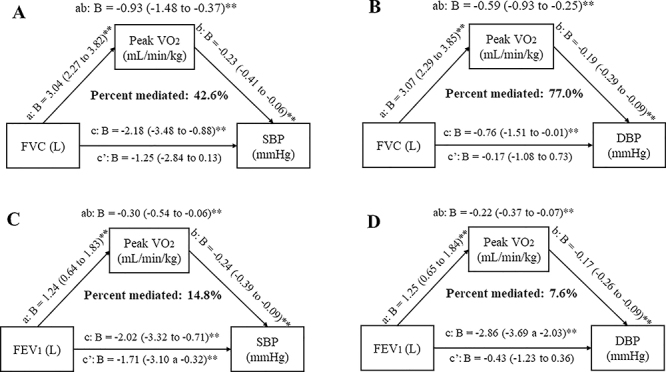
The mediating role of cardiorespiratory fitness (CRF) assessed by the peak oxygen uptake (V̇O_2_) during a treadmill cardiopulmonary exercise testing in the correlation between (**A**) forced vital capacity (FVC) and systolic blood pressure (SBP), (**B**) FVC and diastolic blood pressure (DBP), (**C**) forced expiratory volume in the first second (FEV1) and SBP, and (**D**) FEV1 and DBP. *P<0.05; **P<0.01.

According to the mediation analysis, CRF (i.e., peak V̇O_2_) mediated the relationship between spirometric indices and BP (Figure 4). We found a significant total effect of FVC on SBP and DBP considering the peak V̇O_2_ as a mediator. Indirect effects were also significant, with 42.6% of the total effect of FVC on SBP and 77% of the total effect of FVC on DBP mediated by the peak V̇O_2_. We did not observe a significant direct effect of FVC on SBP and DBP with coefficients different from zero, indicating that this relationship was partially mediated by CRF (Figure 4A and B).

Regarding FEV_1_, the total effect and the indirect effects on SBP were also significant. CRF mediated 14.8 and 7.6% of the relationship between FEV_1_ and SBP and DBP, respectively. However, the direct effects were still significant, indicating a weak partial mediation of the peak *V̇*O_2,_ in the relationship between FEV_1_ and BP variables (Figure 4C and D).

**Figure 5:**
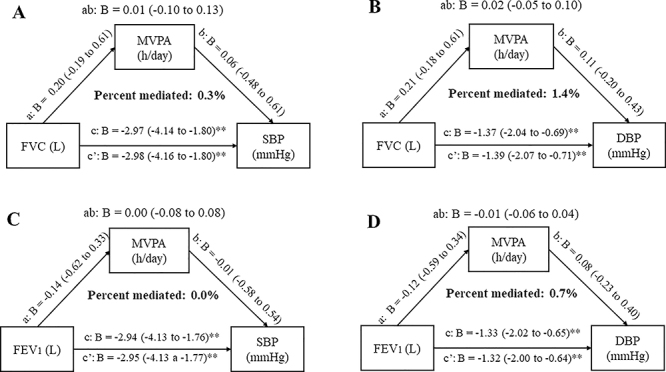
The mediation role of the cardiorespiratory fitness (CRF) assessed by moderate-to-vigorous physical activity (MVPA) during a treadmill cardiopulmonary exercise testing in the correlation between (**A**) forced vital capacity (FVC) and systolic blood pressure (SBP), (**B**) FVC and diastolic blood pressure (DBP), (**C**) forced expiratory volume in the first second (FEV1) and SBP, and (**D**) FEV1 and DBP. *P<0.05; **P<0.01.

As expected, the total and direct effects of FVC and FEV_1_ on BP indices were significant (Figure 5). Nevertheless, MVPA was not considered as a mediator since we did not observe any significant indirect effect of FVC or FEV_1_ on BP.

## Discussion

In the present study, we aimed to investigate whether CRF and MVPA mediated the relationship between spirometric indices and BP in adults. According to our results, CRF is a mediator linking lung function and BP in asymptomatic adults. Unexpectedly, MVPA did not play a mediating role in this relationship.

Robust longitudinal studies have already established the cause-and-effect relationship between low lung function and elevated BP (6,8,19,30). Regarding path diagrams, we observed that both FVC and FEV_1_ presented effects on BP. Also, the models considering FVC as the independent variable showed more significant effects than FEV_1_. Interestingly, Imaizumi et al. (31) found similar results. They evaluated 95 patients with hypertension without previously diagnosed respiratory diseases and showed that the lower the % FVC, the higher the daytime SBP (31). In addition, reduced FVC could inhibit lung stretch receptors and, consequently, activate the sympathetic nervous system, leading to increases in BP (31).

Although the relationship between lung function and hypertension has been suggested previously (19), the pathways underlying this relationship remain unclear and require further research. Moreover, our results indicated that changes in lung function do not directly affect BP due to the mediating role of CRF. It is well known that high peak V̇O_2_ represents better overall health status, while low peak V̇O_2_ is a strong predictor of cardiovascular disease and increased risk of death (9). Exercise training and lifestyle changes lead not only to adaptations in the cardiovascular system, but also to changes in CRF, with little or no effect on lung function (32). However, a recent review showed that lung function declines proportionally to CRF with advancing age (32). Accordingly, a longitudinal study showed that a high CRF in early adulthood is associated with low decline in FVC and FEV_1_ after 20 years, regardless of smoking load (8). We attributed our results to the strong association between lung function and CRF and to the genetic determination of both. While lung function can be genetically determined in about 80% (33), up to 60% CRF can (34). The fact that lung function has an even more significant genetic influence than CRF supports the pathways proposed in the present study. Low lung function probably contributes to a poor CRF and, therefore, indirectly affects BP. Another potential explanation for our results is related to hemodynamic and autonomic aspects. Bianchim et al. (35) showed an association between spirometric indices and autonomic cardiovascular function evaluated using heart rate variability, regardless of smoking and physical inactivity. Additionally, a high CRF indicates a more cardioprotective profile, including better adapted autonomic tone and endothelial function (9).

Contrary to what we initially expected, MVPA was not a significant mediator between lung function and BP. Previously, Jacobs et al. (6) showed that the change in FVC has a consistent relationship with the incidence of hypertension in adults in ten years of follow-up, regardless of physical activity. We can attribute this finding, at least in part, to MVPA being external to body functioning, and hence, a behavioral predictor rather than a mediator itself. In this perspective, MVPA would act more as a confounding variable than as a mediator in the relationship between spirometric indices and BP. Moreover, aerobic training led to more adaptations in the cardiovascular and neuromuscular systems than in lung structure and function (32). Therefore, it seems better to develop strategies to increase CRF to prevent hypertension than to aim at improving lung function, which responds better to medication than to exercise training.

The present study has limitations that should be considered. The study design supposedly precludes the cause-and-effect relationship. However, it is worth noting that the mediation analyses allow the evaluation of underlying pathways linking cause and effect, regardless of study design (13,14). Cardiovascular risk was self-reported, which may have introduced information bias. Lastly, BP measurements were not obtained from a 24-h ambulatory monitoring. Although BP measurements were undertaken during the known blood pressure morning plateau, other variables were obtained throughout the day (i.e., spirometry and CPET). Similar to blood pressure, lung function, heart rate, and cardiorespiratory fitness have diurnal and seasonal variations that must be considered (16,). As these evaluations were carried out during the morning and afternoon, this is another limitation of the present study. Despite a significant diurnal and day-to-day variation, Knaier et al. (39) found that peak V̇O_2_ was not affected by time of day. Participants' characteristics, such as chronotype (36,[Bibr B37] [Bibr B38] 39,40), may play a role in achieving peak V̇O_2_ and/or maximum heart rate. However, we did not consider these aspects in the present study.

In contrast, one of the main strengths of this study was the unprecedented investigation of the role of CRF and MVPA as mediators of the relationship between spirometric indices and BP. To our knowledge, this is the first study to consider CRF as a mediator rather than a confounder, linking lung function and BP. Another strength of this study was the use of direct measurements of both CRF and MVPA, which reduces the information bias commonly associated with estimated peak V̇O_2_ and physical activity assessed by questionnaires. It is worth mentioning that our sample size was robust enough to conduct the proposed analysis.

The key message of our study was that a combination of low FVC and low peak V̇O_2_ might be an alert to incipient elevated BP. Considering the mediating role of CRF between lung function and BP, we believe that CRF should be assessed in clinical practice using the CPET or even field walking tests. Furthermore, mild or moderate changes in lung function are often not diagnosed and hence underestimated. Despite spirometry being neglected in clinical practice, future studies should investigate the contribution that routine spirometry, a simple and inexpensive test, can make to early identification of subjects at increased risk for developing hypertension. In addition, routine assessment of spirometric indices and CRF seems to contribute to the design of more assertive preventive strategies for reducing the incidence of hypertension.

### Conclusion

We may conclude that CRF mediated the relationship between lung function and BP in asymptomatic adults, mainly for FVC. Unexpectedly, MVPA was not a mediator of this relationship. A pathway involving poor CRF should be considered when assessing the impact of lung function on elevated BP. Lastly, we noted that a combination of prophylactic spirometry and CRF assessment may represent a valuable tool for hypertension and cardiovascular risk screening.
